# Band gap maps beyond the delocalization limit: correlation between optical band gaps and plasmon energies at the nanoscale

**DOI:** 10.1038/s41598-017-18949-9

**Published:** 2018-01-16

**Authors:** Wei Zhan, Vishnukanthan Venkatachalapathy, Thomas Aarholt, Andrej Yu. Kuznetsov, Øystein Prytz

**Affiliations:** Department of Physics, Centre for Materials Science and Nanotechnology, University of Oslo, P.O. Box 1048 Blindern, N-0316 Oslo, Norway

## Abstract

Recent progresses in nanoscale semiconductor technology have heightened the need for measurements of band gaps with high spatial resolution. Band gap mapping can be performed through a combination of probe-corrected scanning transmission electron microscopy (STEM) and monochromated electron energy-loss spectroscopy (EELS), but are rare owing to the complexity of the experiments and the data analysis. Furthermore, although this method is far superior in terms of spatial resolution to any other techniques, it is still fundamentally resolution-limited due to inelastic delocalization of the EELS signal. In this work we have established a quantitative correlation between optical band gaps and plasmon energies using the Zn_1−*x*_Cd_*x*_O/ZnO system as an example, thereby side-stepping the fundamental resolution limits of band gap measurements, and providing a simple and convenient approach to achieve band gap maps with unprecedented spatial resolution.

## Introduction

Wurtzite ZnO, with a wide direct band gap (*E*_*g*_) of ~3.3 eV, can be alloyed with rock salt CdO (direct band gap of ~2.3 eV). Incorporation of Cd into ZnO matrix reduces the band gap, resulting in band gap tunability from UV to the visible spectral range^1^, while maintaining superior properties of the direct band gap^2,3^, thereby benefitting device performance^1^. As previous research illustrates, conventional tools such as photoluminescence (PL)^1^, cathodoluminescence (CL)^4^, optical absorption^5^ and X-ray photoelectron spectroscopy (XPS)^6^ are very useful for band gap structure measurement of doped ZnO with high energy resolution. However, they suffered from limited spatial resolution (several microns) and can only reveal one-dimensional band gap structure, thus calling for the application of high resolution techniques.

Monochromated electron energy-loss spectroscopy (EELS)^7,8^ in a scanning transmission electron microscope (STEM)^9^ is highly suited for this purpose. Moreover, aberration correctors in STEM^10^, allowing more electrons to be brought to a focused spot, greatly enhance the signal-to-noise ratio^11–13^. Specifically, over the past decade, these developments have contributed to detailed studies of semiconductor band gaps^14–18^. Most recently, band gap mapping with spatial resolution well below 10 nm has been proved using monochromated EELS in probe-corrected STEM without special setup of the microscope^19^, differing remarkably from the research carried out by Lin Gu *et al*.^16^, who developed energy-filtered STEM to enable the band gap mapping. Experimentally obtained EEL spectra give access to detailed information about the dielectric response, and can be directly compared to theoretical simulations^20,21^.

Despite these advancements, experimental and data processing challenges in EELS band gap analysis^19,22^ hinder wide-spread application of band gap mapping, so that nanoscale band gap measurements are still very rare. Furthermore, the identification of the band gap signal can be complicated by the presence of additional loss mechanisms such as the excitation of surface plasmons, guided light modes, and Cherenkov radiation. Finally, although the spatial resolution realized in the direct mapping of band gap transitions in EELS is far superior to that of other techniques, it is still unfortunately limited by the fundamentals of the scattering process^23^. This means that resolution below the theoretically predicted inelastic delocalization length (*L*_50_) of 5–7 nm is probably not feasible regardless of future technical advances in instrumentation.

In this work, using monochromated EELS in probe-corrected STEM, we investigate the relationship between band gaps and plasmon energies, and establish a robust quantitative correlation using in the ZnO-CdO alloys as an example. This provides a fast and effortless pathway to carry out band gap mapping that can be performed without the need of special hardware such as monochromators or probe Cs correctors, and with much less complexity in the experimental acquisition and data analysis. We furthermore show that using this approach we achieve a higher spatial resolution than the conventional method, without compromising the accuracy.

## Background

In the periodic table, Cd is located directly below Zn and can therefore be considered iso-electronic. However, Cd has a significantly larger ionic radius than Zn, and when Cd^2+^ ions (radius 0.097 nm) replace Zn^2+^ (radius 0.074 nm) in the wurtzite ZnO matrix, the unit cell volume is increased while the band gap is reduced^1,4,24,25^.

Meanwhile, higher Cd content is also associated with a drop in plasmon energy, since the valence electron density decreases as the unit cell volume expands. The plasmons are collective excitations of valence electrons triggered by their collective response to the repulsive field carried by the incident electron. In a free electron model, the plasmon energy is given by^25^:

1 $${E}_{p,F}=\,\hslash {\omega }_{p}=\hslash \sqrt{\frac{N{e}^{2}}{V(x){m}_{0}{\varepsilon }_{0}}}$$

where *E*_*p*,*F*_ is the free electron plasmon energy in EELS spectrum, *ω*_*p*_ is the plasmon frequency, *ħ* is the reduced Planck constant, *N* is the number of valence electrons per unit cell, *e* is the elementary charge, *V*(*x*) is the Cd-concentration dependent volume of unit cell, *m*_0_ is the electron mass, and *ε*_0_ is the permittivity of free space. This free electron model assumes that the valence electrons behave as simple harmonic oscillators, which is an obvious simplification when considering real materials. Nevertheless, many simple metals (e.g. Be, B, Na, Al) and semiconductors (e.g. Si, Ge, GaAs) have sharp plasmon peaks near the value predicted by this model^26^, and it has also been shown that Equation (1) is quite successful in estimating the plasmon energy of more complex materials^21^.

For wide band gap semiconductors, the free electron model can be modified by introducing a bound oscillation with frequency *ω*_*b*_ = *E*_*g*_/*ħ*, so that a semi-free electron plasmon energy can be obtained^25^:

2 $${E}_{p,sF}=\sqrt{{E}_{p,F}^{2}+{E}_{g}^{2}}$$

thereby improving the correspondence with the experimental values somewhat.

As both the band gap and plasmon energy depend on the Cd concentration (*x*), these models predict a relationship between the band gap and the plasmon energy: via the volume alone in the case of the free electron model, while in the semi-free model the band gap energy also appears directly. This indicates that a quantitative connection between the plasmon and band gap energies may be formulated. This theory is elaborated further in the Supplementary Information section 5, and forms the basic justification for the approach taken in the present work.

## Results

### Shifts of *E*_*g*_ and *E*_*p*_ of ZnO after incorporation of Cd

**Figure 1:**
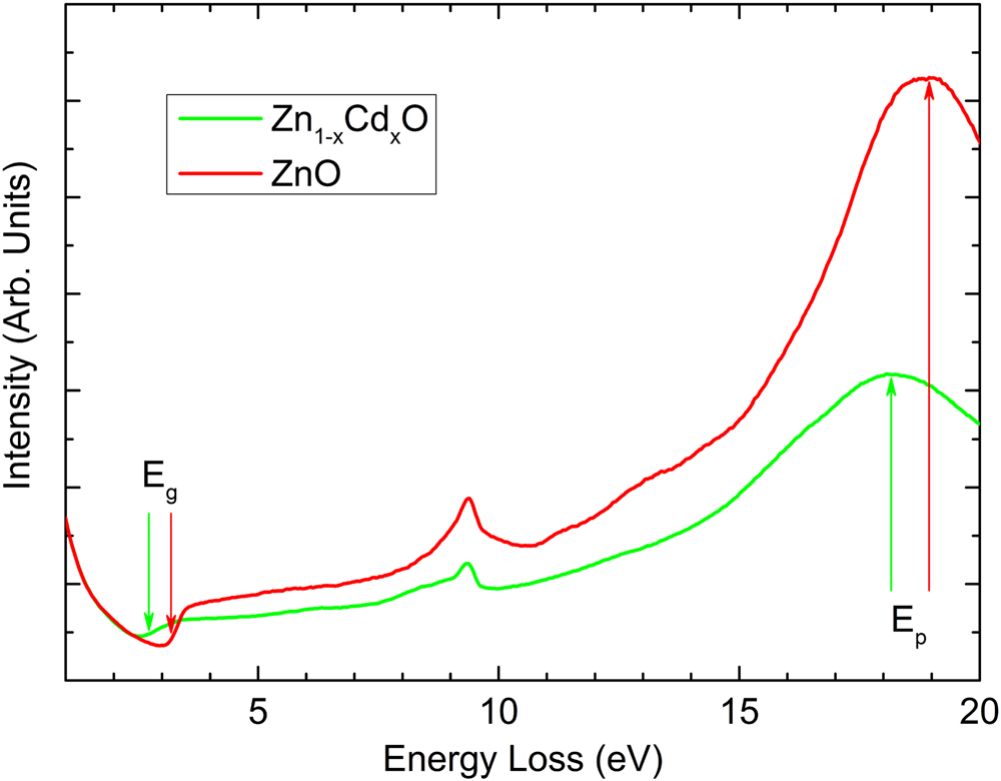
Two single EELS spectra taken from the pure ZnO and Cd-containing layers. Shifts of the plasmon and band gap energies are clearly observed as indicated by arrows.

Figure 1 illustrates experimental spectra obtained from the pure ZnO buffer layer and the alloyed layer with a large amount of Cd. Both the plasmon peaks and the band gap energy loss onsets are easily identified. To extract the band gap value, a power-law model was used to reliably subtract the background signal at energies *E* < *E*_*g*_, and a parabola was fitted to the remaining spectrum, further details about this procedure can be found elsewhere^18,19^. In pure ZnO, the average band gap is found to be 3.22 ± 0.02 eV using the fitting range 2.4–2.9 eV for background subtraction (Supplementary Fig. S8a), in agreement with previous investigations^19^. In order to measure the plasmon energy, we first employed the Fourier-log method, as implemented in DigitalMicrograph, to remove plural scattering. Thereafter the EELS spectra within a selected narrow energy range were fitted with a Gaussian function to determine the plasmon peak positions. The average plasmon energy of ZnO is 18.88 ± 0.02 eV (Supplementary Fig. S1), consistent with previous reports^27,28^. In comparison, the free electron model (Equation (1)) predicts a theoretical value of 18.64 eV, while the semi-free model (Equation (2)) using the observed band gap as an input leads to a higher value of 18.92 eV. Thus, the two models, and the semi-free model in particular, are in good agreement with the experimental observation. For the Cd-containing layer, Fig. 1 shows that both *E*_*g*_ and *E*_*p*_ are shifted to lower energies, as expected from the unit cell volume expansion caused by the incorporation of Cd atoms into the structure.

### Simultaneous *E*_*g*_ and *E*_*p*_ maps

**Figure 2:**
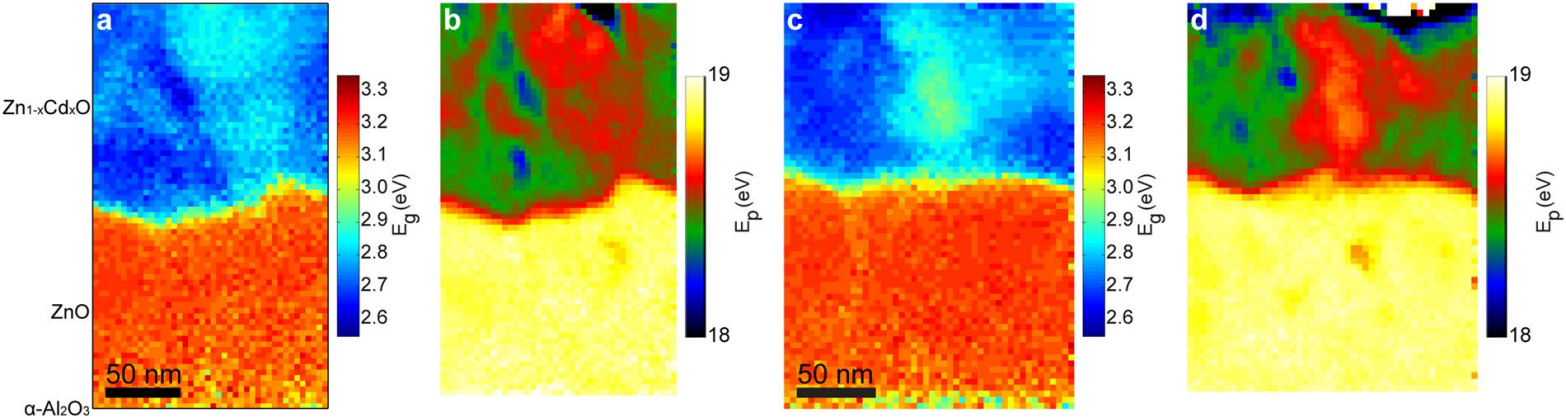
Directly measured (**a**) band gap and (**b**) plasmon energy maps of Zn_1−*x*_Cd_*x*_O/ZnO/α-Al_2_O_3_. Directly measured (**c**) band gap and (**d**) plasmon energy maps from a different region. Zn_1−*x*_Cd_*x*_O was grown on the α-Al_2_O_3_ substrate with a ZnO buffer layer in between.

Figure 2 shows band gap and plasmon energy maps of Zn_1−*x*_Cd_*x*_O/ZnO taken from two different areas. Red and yellow colors indicate a high band gap or plasmon energy, while blue and green imply a lower value. As demonstrated by energy-dispersive X-ray spectroscopy (EDX) maps obtained for Cd and Zn (see Supplementary Figs S2 and S3), the film exhibits the expected two-layer structure, with an inner buffer layer consisting of pure ZnO, and an outer layer of Cd-containing ZnO^19^. The α-Al_2_O_3_ substrate is situated at the bottom. The thickness of each layer is about 120 nm. The transition from ZnO to the Zn_1−*x*_Cd_*x*_O layer is clearly visible as a rather abrupt drop in *E*_*g*_ or *E*_*p*_ values. However, the interface between ZnO and Zn_1−*x*_Cd_*x*_O is rough. Furthermore, we observed that there are significant spatial variations of band gaps and plasmon energies internally in the Cd-containing layers. Intriguingly, their variations match very well, and are supported by the elemental EDX maps in Supplementary Information. These EDX measurements confirm that the higher Cd content is correlated with decreasing band gap and plasmon energy, and that the maximum Cd content (*x*) in our specimens is ≈0.51. Previous work^2^ shows that Zn_1−*x*_Cd_*x*_O stabilizes single-phase wurtzite with Cd content *x* up to 0.67, this is also confirmed by our X-ray diffraction investigations.

In comparison, the variations observed in Fig. 2 within the pure ZnO layer are much smaller, suggesting a high spectral precision. Note that the optical band gaps and plasmon energies were simultaneously acquired pixel by pixel. Therefore these maps with point-to-point correspondence are well suited to investigate their relationship and establish a quantitative correlation. Importantly, the plasmon energy map displays a significantly better spatial resolution than the band gap map, and there are some small energy variations that are undetectable in the band gap map. This is expected from the difference in EELS spatial resolving ability, which depends on the energy loss as well as high tension of the microscope. As an example, in the current experimental setup with 60 keV incident electrons, the EELS spatial resolution as expressed by the inelastic delocalization length (*L*_50_) is estimated to be approximately 5.41 nm for the band gap transitions in ZnO, and 1.46 nm in the case of its plasmon excitations^23,25,29^. See the Supplementary Information for further details.

### Quantitative correlation between *E*_*g*_ and *E*_*p*_

**Figure 3:**
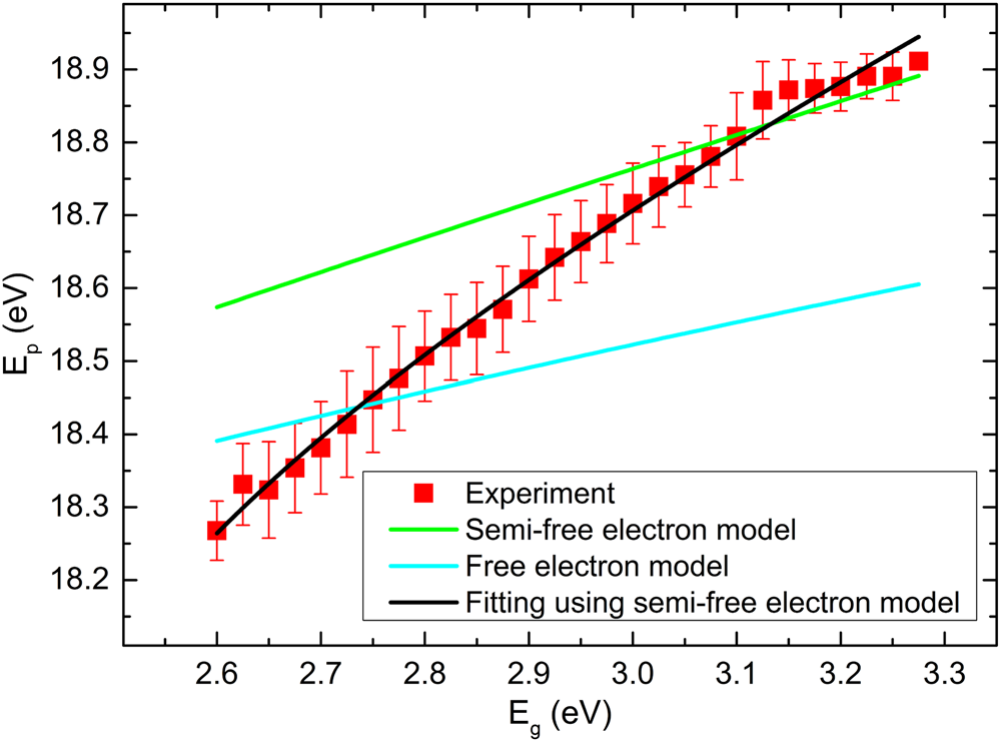
The observed *E*_*p*_ – *E*_*g*_ correlation plotted together with the values predicted from the free and semi-free electron models based on literature inputs. The fitting of the semi-free electron model to the experimental data is plotted in black.

To establish a quantitative relationship between the plasmon energy and band gap, several experiments such as those in Fig. 2 were performed on different regions of the sample. In Fig. 3 we have plotted the observed band gap and plasmon energy in 13876 pixels (spectra) originating from eleven different spectrum images, sufficient for establishing the quantitative correlation. Owing to the poorer spatial resolution of the band gap map, several different plasmon energies may be observed for each particular value of the band gap. This is indicated by the error bars which show the spread (one standard deviation) of plasmon energies around the average value.

As shown in the Supplementary Information a relationship between the plasmon energy and band gap can be derived. In the free electron model this relationship is as follows:

3 $${E}_{p,F}=a\ast \sqrt{\frac{2bg-cf+\sqrt{4{E}_{g}{c}^{2}g-4{c}^{2}eg+{c}^{2}{f}^{2}}}{2({b}^{2}g+{c}^{2}e-bcf-{E}_{g}{c}^{2})}}$$

while for semiconductor or insulator the semi-free model should be used, resulting in the following equation:

4 $${E}_{p,sF}=\sqrt{{{E}_{g}}^{2}+{a}^{2}\frac{2bg-cf+\sqrt{4{E}_{g}{c}^{2}g-4{c}^{2}eg+{c}^{2}{f}^{2}}}{2({b}^{2}g+{c}^{2}e-bcf-{E}_{g}{c}^{2})}}$$

Here, *a*, *b*, *c*, *d*, *e*, *f*, *g* and *N* are all constants that can be found in literature, see Table 1 for an overview.

**Table 1:** Fitting parameters for experimental data and Eq. 4.

Parameter	Literature value	Fitted value (semi-free model)	Comment/Reference
*a*	128.566	128.4767	From free electron plasmon model, see Equation (S3)
*b*	47.6093	47.6093	ZnO unit cell volume. Reference^24^
*c*	4.14502	4.139997	Reference^24^
*d*	0	—	Reference^24^
*e*	3.37	3.215	Band gap of pure ZnO. Reference^3^
*f*	−2.82	−1.194	Band bowing parameter. Reference^3^
*g*	0.95	0.45	Band bowing parameter. Reference^3^
*N*	12	11.98	Valence electron numbers in ZnO unit cell are used to calculate literature value of *a*. The fitted value 11.98 is calculated from the fitted value of *a*.

The relationship predicted between *E*_*g*_ and *E*_*p*_ predicted by the free and semi-free electron models using the literature inputs from Table 1 are plotted in Fig. 3. It can be seen that the two models are somewhat successful in estimating the plasmon energy in the low gap and high gap range respectively, but neither of the models offer satisfactory results over the entire range.

We now follow two different routes to establish the quantitative relationship between the band gap and plasmon energy. First a polynomial function relating *E*_*g*_ and *E*_*p*_ is fitted on the basis of the experimental data, as shown in Supplementary Fig. S5. Although this results in a rather exact fit, it does not directly relate to any of the physical parameters that serve as determining factors in the variation of band gap and plasmon energy. Instead, we take Equation (4) above as a starting point, and use the constants as fitting parameters, arriving at a correlation described by the black line in Fig. 3 and the parameters in Table 1. An excellent match with the observed correlation can be achieved, while at the same time retaining physically realistic and meaningful fitting parameters. It is particularly encouraging that reasonable values of the unit cell volume and the band gap are kept. It needs to be pointed out that the Cd compositional range in our work differs significantly from the literature^3^, resulting in a significant discrepancy between the fitted and the literature values of *f* and *g*.

### Reconstructed *E*_*g*_ map with improved spatial resolution

**Figure 4:**
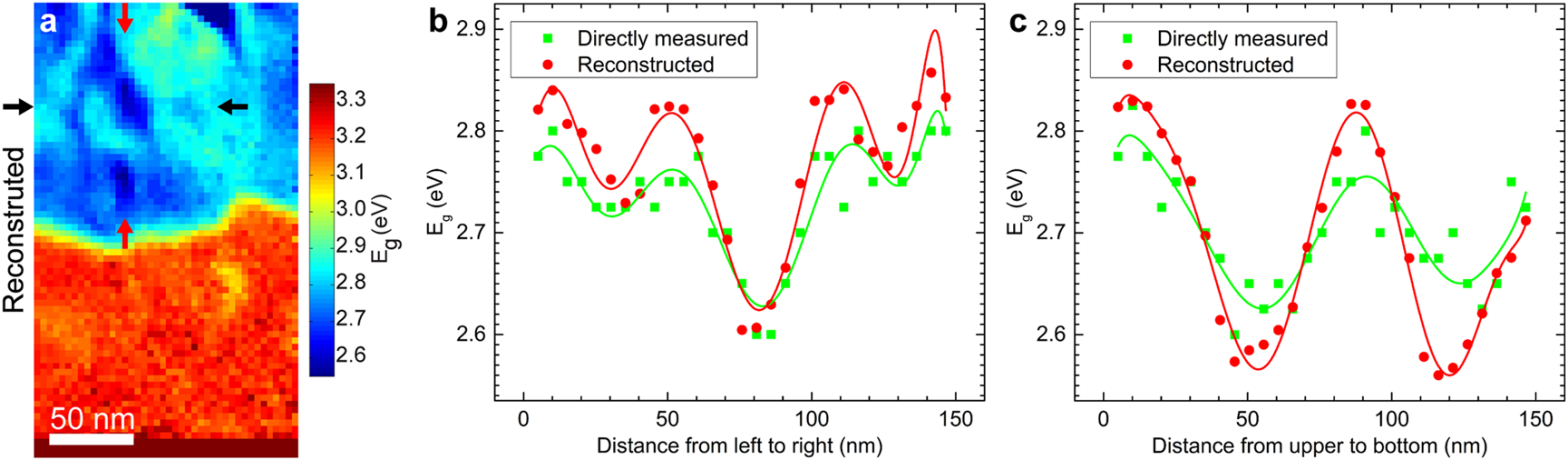
(**a**) Band gap map reconstructed from the plasmon energy map (Fig. 2b) using the semi-free electron fitting. The arrows display the start and end points of the two lines chosen for analysis in (**b**), (**c**). Directly measured (Fig. 2a) and reconstructed *E*_*g*_ along the horizontal (**b**) and vertical (**c**) line profiles. Polynomial curves are superimposed to guide the eyes.

**Figure 5:**
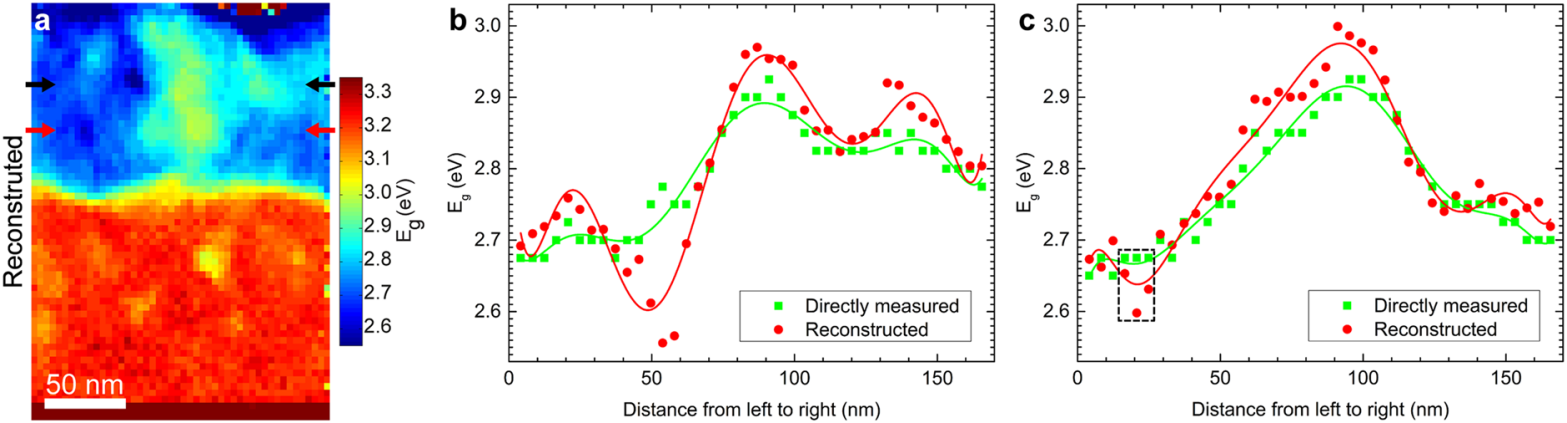
(**a**) Band gap map reconstructed from the plasmon energy map (Fig. 2d) using the semi-free electron fitting. The arrows display the start and end points of the two lines chosen for analysis in (**b**), (**c**). Directly measured (Fig. 2c) and reconstructed *E*_*g*_ along the top (b) and bottom (c) horizontal line profiles. Polynomial curves are superimposed to guide the eyes.

The proposed relationship between the plasmon energy and band gap can now be employed to reconstruct band gap maps from plasmon energy maps. It was not possible to acquire an analytical solution of Equation (4) for *E*_*g*_ in terms of *E*_*p*_. Instead, the equation was solved numerically by slowly increasing *E*_*g*_ until a value equal or larger than *E*_*p*_ was found, for each pixel of a plasmon energy map. See the Supplementary Information for attached python code. This was applied to the two data sets shown in Fig. 2. The resulting reconstructed band gap maps are shown in Figs 4a and 5a. For convenience, the color scale here remains the same as the directly measured *E*_*g*_ map in Fig. 2a,c. As expected, the directly measured and reconstructed maps show a strong similarity, but the reconstructed map clearly resolves several additional variations not observable in the directly measured *E*_*g*_ maps. Line profiles from the reconstructed maps are shown in Figs 4b,c and 5b,c together with the corresponding line profiles from the directly measured maps as indicated by red and black arrows. These line profiles confirm that a greater resolution is achieved in the reconstructed maps.

Furthermore, as shown in Supplementary Fig. S8a,b, the reconstructed band gaps in the chemically homogeneous ZnO layer are very close to the directly measured values. By averaging over 260 pixels an average value of the reconstructed band gap of 3.24 ± 0.02 eV is obtained compared to the directly measured value of 3.22 ± 0.02 eV, thereby showing that a good accuracy is retained. Note that, in Figs 4b,c and 5b,c, the band gap values are revealed by the data points, and the polynomial curves are just added as a guide to the eye. Both the directly measured and reconstructed maps exhibit some standard deviations, which causes the shifts of the maximum (or minimum) position of the polynomial curves.

## Discussion

Compared to the directly measured band gap maps, the reconstructed maps have significantly higher spatial resolution. As shown in Figs 4 and 5, the two methods capture the same general features, but the reconstructed map reveal both higher and lower absolute values. There are two effects contributing to this difference. First, in the case of the directly measured band gap maps, the incident electron not only experience energy transfer to excitations “locally”, but also to transitions taking place some distance away from the position where it penetrates the specimen. This inelastic delocalization of the signal causes a spectrum from one position on the sample to have contributions from a larger volume, quantified as an inelastic delocalization length (*L*_50_, contributing 50% of the signal) of approximately 6 nm (see Supplementary Information for more details). Second, to create the band gap map, the experimental spectra need to be analyzed in order to identify the onset of energy loss corresponding to the band gap. In this process, even small or moderate contributions from adjacent areas can weigh heavily and thereby causing a broadening of the features^19,22^. For a feature to be resolved using this direct mapping method, it must therefore be quite large both spatially and spectrally.

These problems are greatly reduced when using the *E*_*p*_-to-*E*_*g*_ reconstruction approach. First, the inelastic delocalization is much smaller in the case of plasmon losses, and second, the data processing now only requires determination of the plasmon peak position, a procedure that is much less influenced by contributions from adjacent areas than attempting to find an energy loss onset. The improvements in resolution are clearly evident in the finer details of Fig. 5. Here several features that were not resolved in the directly measured maps now become apparent. For example, in Fig. 5c (dashed box) we see a sharp drop in the band gap between adjacent points in the reconstructed data. The distance between these points is only 4.14 nm, while the *E*_*g*_ reduction is well above the statistical error. In comparison, this feature is completely missing in the directly measured data.

As mentioned in Supplementary Information section 3, the spatial resolution of the directly measured band gaps could in theory be improved by using a different experimental setup. If a specific spatial resolution is required for a given excitation energy, the resolution could in principle be improved by lowering the accelerating voltage of the microscope. However, as shown in Supplementary Fig. S4, a dramatic reduction in accelerating voltage is needed to reduce the inelastic delocalization to comparable levels as that predicted for plasmon losses at 19 eV: an *L*_50_ of 1.46 nm is achieved for 3 eV band gaps only by reducing the accelerating voltage below 0.18 kV. While monochromatic electron beams with such low energy can indeed be generated, the increase in beam size (and resulting reduction in resolution) far outweighs the improvement in inelastic delocalization. In comparison, the *E*_*p*_-to-*E*_*g*_ reconstruction approach can be used on regular samples at standard accelerating voltages, while still achieving excellent spatial resolution, accuracy and precision.

## Conclusions

In summary, taking advantage of state-of-the-art monochromated EELS in conjunction with probe-corrected STEM, the local optical band gaps and plasmon energies of Zn_1−*x*_Cd_*x*_O/ZnO were simultaneously mapped on the nanometer scale with a high level of spectral precision, and their quantitative correlation was successfully established. This provides a practical method to acquire semiconductor band gap values via plasmon energies, with drastically improved spatial resolution as compared to the direct measurement of the band gap. These findings pave the way for studies of band gap engineered semiconductor nanostructures with spatial resolution beyond the traditional delocalization limits, with the added benefit of greatly relaxed requirements on hardware and data processing.

## Methods

Thin-film specimen of Zn_1−*x*_Cd_*x*_O with variable Cd concentrations was synthesized by metal organic vapour phase epitaxy (MOVPE) on α-Al_2_O_3_ substrate along the [0001] axis buffered with a pure ZnO layer. The sample for STEM investigations was prepared by cutting the slices along the [0001] direction of α-Al_2_O_3_. These slices were then mechanically ground to 150 μm, after which the slices were made into wedges with a tilt angle of 2.5°, using the MultiPrep System (Allied High Tech Products, USA). One side of the slices was further ground down to 20 μm. Thereafter the ground side was thinned by the low-angle ion milling & polishing system (Fischione 1010) with gun voltages of 5 kV/4 kV/3 kV, gun currents of 5 mA/4 mA/3 mA, and an incident beam angle of 8°. The total milling time was about 3 h.

Immediately before the STEM experiments were performed, the sample was plasma cleaned in a Fischione Model 1020 plasma cleaner. The STEM investigations were undertaken in a probe-corrected and monochromated FEI Titan G2 60–300, equipped with a Fischione HAADF detector (3000), a Gatan GIF Quantum 965 EELS spectrometer, and four FEI Super-X EDX detectors. The actual content of Cd in Zn_1−*x*_Cd_*x*_O was studied by EDX maps at a 300 kV accelerating voltage. Zn Kα and Cd Lα characteristic X-ray peaks were used for EDX quantification of content, and Cd content was determined to be 0.01 < *x* < 0.51. To prevent the Cherenkov radiation from overlapping with the band gap, monochromated EELS spectrum imaging were operated at a high tension of 60 kV, close to Cherenkov limit of ZnO. The specimens were finally thinned to be approximately 20~30 nm, further efficiently eliminating the unwanted retardation losses. The dispersion of the 2048-channel EELS spectrometer was set at 0.025 eV/channel in order to simultaneously collect the signals of band gap and plasmon energy pixel by pixel. To guarantee sufficient signals for EELS spectrum image, the time for exposure at each pixel was very close to the limiting exposure for the CCD. Before carrying out the structural and spectral mapping, the α-Al_2_O_3_ substrate was tilted to the [2 $\bar{1}\bar{1}\,$0] orientation to make the electron beam perpendicular to the film growth direction. During the EELS or EDX spectrum imaging, software correction of the spatial drift was employed.

The energy resolution in the monochromated EELS measurements was approximately 0.175 eV, as determined by the full width at half maximum (FWHM) of the zero-loss peak (ZLP). The band gap is identified as the onset of energy loss (similar to the onset of absorption in optical absorption experiments) followed by fitting a parabolic model to the spectrum after background subtraction. An EELS spectroscopic image is composed of many pixels (spectra). The parabolic fitting performed at each spectrum would eventually lead to a two-dimensional band gap image. A detailed explanation of the steps taken for band gap fitting has been published previously^19,22^. The plasmon energy values in EELS spectrum were obtained by fitting a Gaussian function in DigitalMicrograph, before which plural scattering was removed using the Fourier-log method.The datasets generated during and/or analyzed during the current study are available from the corresponding author upon a reasonable request.

## Electronic supplementary material

Supplementary information
