# Supplementary material for: Band gap maps beyond the delocalization limit: correlation between optical band gaps and plasmon energies at the nanoscale
Source: Sci Rep. 2018 Jan 16;8:848. doi: 10.1038/s41598-017-18949-9 (PMC5770386; doi:10.1038/s41598-017-18949-9)
Supplement: Supplementary file 1 — Supplementary information [file 41598_2017_18949_MOESM1_ESM.pdf]

Supplementary Information

# Band gap maps beyond the delocalization limit: correlation between optical band gaps and plasmon energies at the nanoscale

**Wei Zhan, Vishnukanthan Venkatachalapathy, Thomas Aarholt, Andrej Yu. Kuznetsov, and Øystein Prytz***

Department of Physics, Centre for Materials Science and Nanotechnology, University of Oslo, P.O. Box 1048 Blindern, N-0316 Oslo, Norway

*E-mail: oystein.prytz@fys.uio.no

## 1. Determination of ZnO plasmon energy

As shown in Supplementary Fig. S1, employing monochromated EELS in combination with probe-corrected STEM, the average plasmon energy found for the pure ZnO layer is approximately 18.88 eV by extracting from 260 pixels, and their standard deviation (σ) is typically 0.02 eV. The plasmon energy is in good agreement with previous measurements [1, 2].

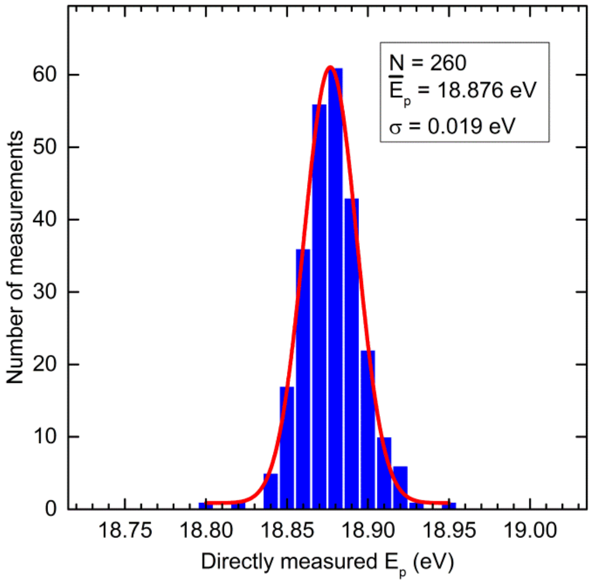


**Supplementary Figure S1.** Histogram of the plasmon energy values as extracted from the pure ZnO buffer layer, superimposed by a Gaussian fitting.

## 2. EDX maps of the $E_g$ and $E_p$ imaging areas

Supplementary Figure S2 illustrates annular dark-field (ADF) STEM image and EDX maps of $Zn_{1-x}Cd_xO$/ZnO observed from the same position as band gap and plasmon energy imaging in Fig. 2a,b (main text). By comparison, a conclusion can be drawn: the decrease (rise) of band gap or plasmon energy in the Cd-containing layer is correlated with the rise (drop) of Cd content $x$. The α-$Al_2O_3$ substrate is located at the bottom, and in the plasmon maps (Figs. 2b and 2d main text), the color is white (not visible) since its value (>19 eV) is out of the color range chosen for $E_p$ in $Zn_{1-x}Cd_xO$/ZnO. Note that the band gap and plasmon energy variations in the α-$Al_2O_3$ substrate are outside the scope of this study.

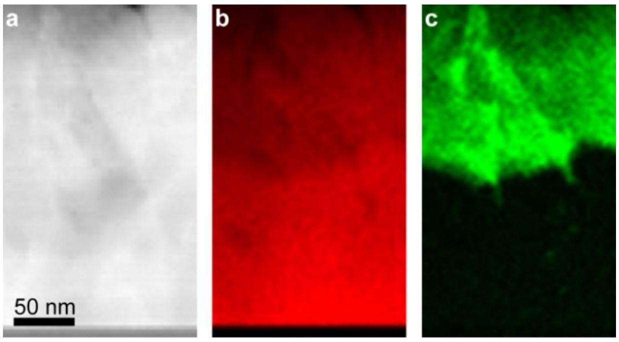

**Supplementary Figure S2.** (a) ADF image of $Zn_{1-x}Cd_xO/ZnO/\alpha\text{-}Al_2O_3$. EDX maps of (b) Zn and (c) Cd from the same area of Fig. 2a,b (main text).

Supplementary Figure S3 displays ADF image and EDX maps of $Zn_{1-x}Cd_xO/ZnO$ taken from the same position as Fig. 2c,d (main text). Cd content changes here are consistent with the variations in band gap and plasmon energy.

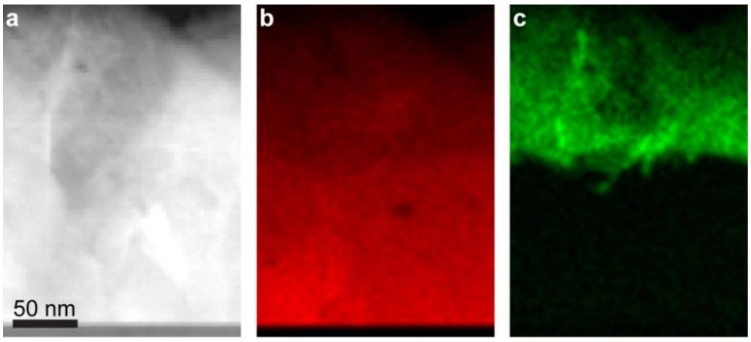

**Supplementary Figure S3.** (a) ADF image of $Zn_{1-x}Cd_xO/ZnO/\alpha\text{-}Al_2O_3$. EDX maps of (b) Zn and (c) Cd from the same region of Fig. 2c,d (main text).

## 3. EELS spatial resolution

It has been mentioned in main text that experimental and analytical difficulties remain a barrier to the wide-spread adoption of STEM-EELS band gap mapping [3, 4]. The setup of hardware required, especially monochromator, is accounted for the complexity in performing experiment. It is known that monochromators can greatly improve energy resolution by energy-filtering the incident beam. However, it also significantly reduces the beam current and thereby the observable signal. Lin Gu and co-authors [5] proposed energy-filtered STEM to improve the signal collection and thus make band gap mapping possible. In comparison, there is no special instrumental design required in this work (nor in our previous research [3, 4]). In fact, we set the exposure time of each pixel close to the limiting exposure for the CCD. In addition, the thin sample, long-time acquisition and good adjacent environment also helped the measurements.

The factors that affect EELS spatial resolution are described below. In previous work [6-8] it has been reported that the spatial resolution is determined by the inelastic delocalization length

$$L_{50} = \frac{0.44hc_0[eU(eU + 2m_0{c_0}^2)]^{\frac{1}{4}}}{[E(eU + m_0{c_0}^2)]^{\frac{3}{4}}} \tag{S1}$$

where $L_{50}$ is the diameter containing 50% of the inelastic scattering events, $h$ is the Planck constant, $c_0$ is light speed, $e$ is elementary charge, $U$ is accelerating voltage, $m_0$ is electron

mass, $E$ is energy loss.

As can be seen from Equation (S1), the spatial resolution ($L_{50}$) depends on energy loss ($E$) and accelerating voltage ($U$). The spatial resolution could therefore in theory be improved by reducing the accelerating voltage of the microscope. However, for a particular energy loss, even quite dramatic reductions in accelerating voltage have only a modest effect on the spatial resolution, which limits the usefulness of this approach. To exemplify this we have plotted $L_{50}$ for two relevant energy losses (19 eV and 3 eV) as a function of microscope accelerating voltage. As shown in Supplementary Fig. S4, for a direct measurement of a band gap edge at 3 eV to reach the same level of inelastic delocalization as a plasmon loss of 19 eV, the accelerating voltage would have to be reduced e.g. from 60 kV to 0.18 kV.

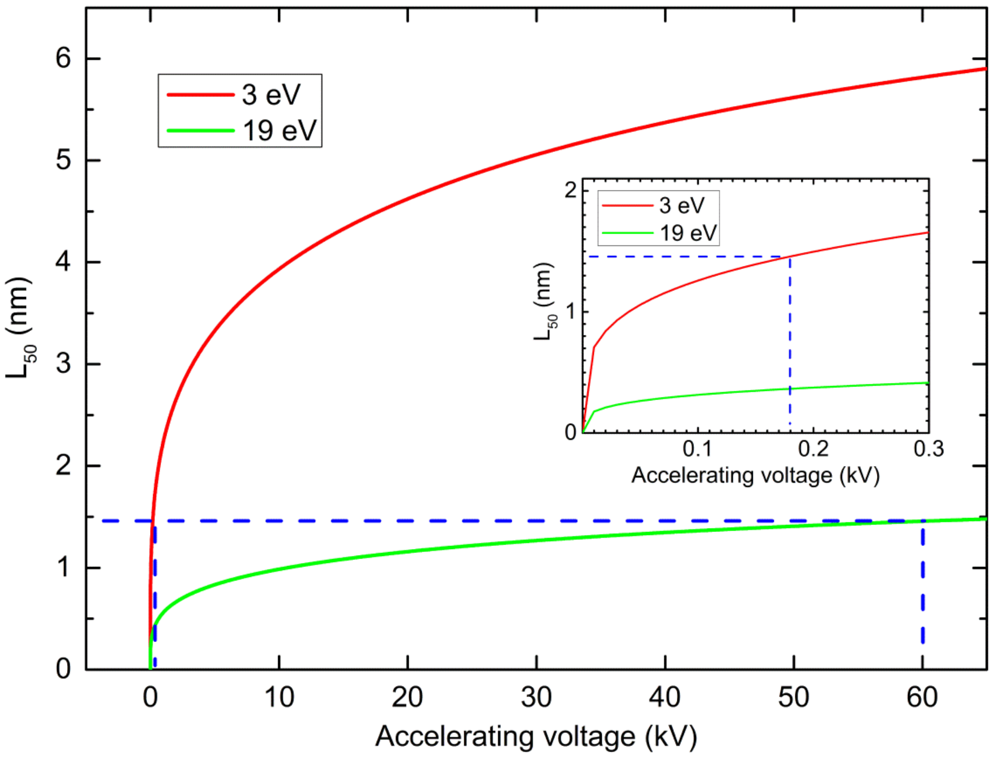


**Supplementary Figure S4.** The inelastic delocalization length $L_{50}$ plotted as a function of microscope accelerating voltage for two relevant energy losses. The inset shows a closeup of the bottom left part of the plot. In order to achieve similar delocalization for a directly measured band gap at 3 eV, the accelerating voltage would have to be reduced to 0.18 kV.

## 4. Quantifying the correlation between $E_p$ and $E_g$ using polynomial function

There is a clear correlation between the observed plasmon energies and band gaps, see Supplementary Fig. S5. To quantitatively relate the two, a simple polynomial function can be fitted to the experimental data as follows.

$$E_p = A + B * E_g + C * {E_g}^D \quad \text{(S2)}$$

where $A$, $B$, $C$ and $D$ are fitting parameters, with the resulting values listed in Supplementary Table S1. The fitting of the experimental data adopting Eq. (S2) is displayed in Supplementary Fig. S5. We find that the polynomial gives a good fit to the experimental data. However, when using such a polynomial function, we cannot expect the fitting parameters to correspond to any physical properties of the material. In the main text we have therefore chosen a physical model for the correlation, which is derived in the next section.

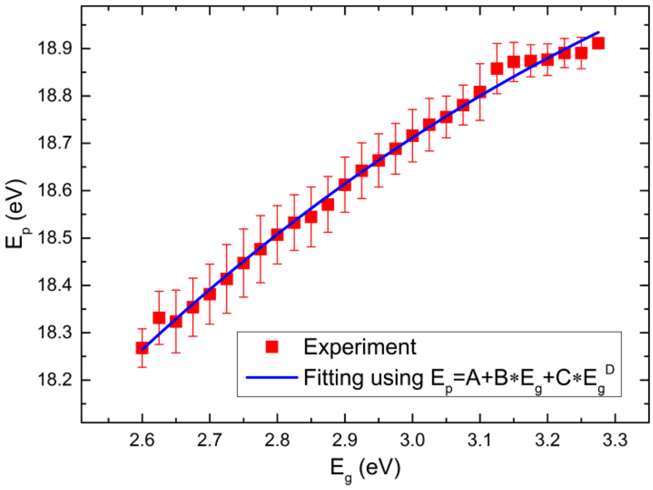


**Supplementary Figure S5.** Fitting the experimental data using Equation (S2).

| Constant | Fitting values |
| --- | --- |
| *A* | -0.859843 |
| *B* | -4.23272 |
| *C* | 19.0521 |
| *D* | 0.479655 |

**Supplementary Table S1.** Parameters in the polynomial fitting equation of the experimental data.

## 5. Free and semi-free electron models for plasmon oscillations

Instead of a polynomial fitting as used in the previous section, we can adopt the two well-known models to provide a fitting function. In the free electron model, for solids with loosely bound outer-shell electrons, the frequency of resonant oscillation is $\omega_p$, giving the free electron plasmon energy [7]

$$E_{p,F} = \hbar\omega_p = \hbar\sqrt{\frac{Ne^2}{V(x)m_0\varepsilon_0}} = \frac{a}{\sqrt{V(x)}} \qquad \text{(S3)}$$

with $a = \hbar\sqrt{\frac{Ne^2}{m_0\varepsilon_0}}$. For ZnO ($x = 0$) with a unit cell volume $V = 47.6093$ Å$^3$ and $N = 12$ valence electrons per unit cell (2 Zn atoms with 8 electrons and 2 O atoms with 4 electrons), a free electron plasmon energy $E_{p,F} = 18.64\ eV$ is obtained.

If ZnO is alloyed with Cd, forming $Zn_{1-x}Cd_xO$, the volume of the unit cell can be described as a polynomial

$$V(x) = b + cx + dx^2 \qquad \text{(S4)}$$

We will now assume $d = 0$ in accordance with Vegard's law, and fit $b$ and $c$ to the linear relationship found by Ghosh, et al [9]. The combination of Equations (S3) and (S4) gives the free electron plasmon energy as a function of composition $x$:

$$E_{p,F} = \frac{a}{\sqrt{b + cx}} \qquad \text{(S5)}$$

The main emphasis of this research lies in the correlation between band gap and plasmon energy, and Wang's band bowing formula as follows can be used [10].

$$E_g(x) = e + fx + gx^2 \tag{S6}$$

Combining Equations (S5) and (S6) we arrive at the set of four equations:

$$E_{p,F} = -a * \sqrt{\frac{2bg - cf - \sqrt{4E_g c^2 g - 4c^2 eg + c^2 f^2}}{2(b^2 g + c^2 e - bcf - E_g c^2)}} \tag{S7a}$$

$$E_{p,F} = a * \sqrt{\frac{2bg - cf - \sqrt{4E_g c^2 g - 4c^2 eg + c^2 f^2}}{2(b^2 g + c^2 e - bcf - E_g c^2)}} \tag{S7b}$$

$$E_{p,F} = -a * \sqrt{\frac{2bg - cf + \sqrt{4E_g c^2 g - 4c^2 eg + c^2 f^2}}{2(b^2 g + c^2 e - bcf - E_g c^2)}} \tag{S7c}$$

$$E_{p,F} = a * \sqrt{\frac{2bg - cf + \sqrt{4E_g c^2 g - 4c^2 eg + c^2 f^2}}{2(b^2 g + c^2 e - bcf - E_g c^2)}} \tag{S7d}$$

These four branches are plotted in Supplementary Fig. S6. Only the fourth branch, corresponding to Equation (S7d), provides physical results with $0 \le x \le 1$. Solving instead for $E_g$ the expression below is obtained:

$$E_g = g\left(\frac{a^2}{{E_p}^2 c} - \frac{b}{c}\right)^2 + f\left(\frac{a^2}{{E_p}^2 c} - \frac{b}{c}\right) + e \tag{S8}$$

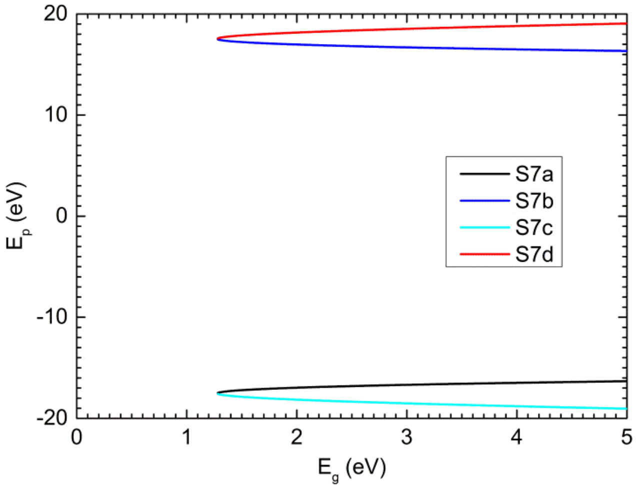


**Supplementary Figure S6.** The four branches (S7a, S7b, S7c, S7d) plotted together.

The free electron model assumed in Equation (S3) will underestimate the observed plasmon energy in semiconductors with a wide band gap. For semiconductor or insulator, a modified version of the plasmon energy takes into account bound electrons with a resonance frequency

$\omega_b$. Setting $\hbar\omega_b = E_g$ brings about the semi-free plasmon energy [7]:

$$E_{p,sF} = \sqrt{E_{p,F}^2 + E_g^2} = \sqrt{\left(\hbar\sqrt{\frac{Ne^2}{V(x)m_0\varepsilon_0}}\right)^2 + E_g^2} = \sqrt{\frac{a^2}{V(x)} + E_g^2} \tag{S9}$$

Using a band gap energy $E_g = 3.22\ eV$, this model leads to a plasmon energy $E_{p,sF} = 18.92\ eV$, which is closer to the experimentally observed value of $E_{p,exp} = 18.88 \pm 0.02\ eV$ than the free electron model.

Further, to obtain plasmon energy as a function of band gap, we proceed in the same manner as before. There are four solutions, only one of which gives physically meaningful results:

$$E_{p,sF} = \sqrt{{E_g}^2 + a^2\frac{2bg - cf + \sqrt{4E_gc^2g - 4c^2eg + c^2f^2}}{2(b^2g + c^2e - bcf - E_gc^2)}} \tag{S10}$$

where $a$, $b$, $c$, $d$, $e$, $f$, $g$, $N$ are all constants, as summarized in Table 1 (main text).

In Supplementary Fig. S7, the two models are plotted together.

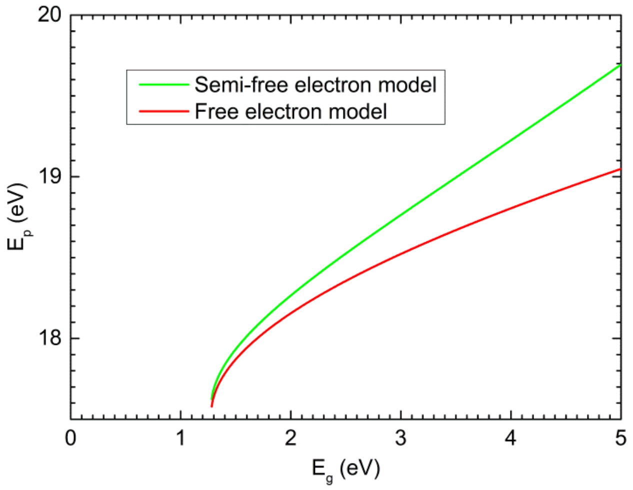


**Supplementary Figure S7.** Free electron model and semi-free electron model plotted together.

## 6. Numerical conversion of $E_p$ to reconstructed $E_g$

The fitted $E_p$-$E_g$ equation adopting semi-free electron model is a monotonically increasing function within the range of $E_g$ (from minimum to maximum), as can also be seen from the fitting curve in Fig. 3 (main text). This implies that there is only one band gap value for a particular plasmon energy, based on which the reconstructed band gap can be extracted. The code that is employed to process $E_p$-to-$E_g$ conversion maps is given below.

```
import hyperspy.api as hs
import numpy as np
import matplotlib.pyplot as plt
import os

path = r"M:\pc\Dokumenter\Notebooks\EpEg"
path = os.path.join(path, "")
name = "Plasmon Map.dm3"
```

```
s = hs.load(path + name)
### Settings constants for the relation Eg -> Ep
a = 128.476702120452
b = 47.6093
c = 4.13999650371382
e = 3.21530501740307
f = -1.19423330394094
g = 0.450088043947096

constants = a,b,c,e,f,g

def Eg_to_Ep(Eg, constants):
    import math
    a,b,c,e,f,g = constants
    return math.sqrt(
        Eg**2 + a**2*(2*b*g-c*f+math.sqrt(4*Eg*c**2*g-4*c**2*e*g+c**2*f**2))
        /(2*(b**2*g+c**2*e-b*c*f-Eg*c**2)))

Ep_signal = s
Eg_signal = s.deepcopy()

Ep = Eg_signal.data

Egvalues = np.arange(2.5,76,0.001)
EgtoEpConversionArray = np.array([Eg_to_Ep(i, constants) for i in Egvalues])

ourEgvalues = Egvalues[EgtoEpConversionArray < 19.5]
ourEpvalues = EgtoEpConversionArray[EgtoEpConversionArray < 19.5]

def func(Epvalue, Epvalues, EpEgConversionArray):
    import numpy as np
    Egvalue = Egvalues[np.where(EgtoEpConversionArray >= Epvalue)[0][0]]
    return Egvalue

for x in np.nditer(Ep, op_flags=['readwrite']):
    x[...] = func(x, Egvalues, EgtoEpConversionArray)

# Save as tif
Eg_signal.save(path + "Eg_values.tif")
# Save for Matlab
import scipy.io
scipy.io.savemat(path+"Eg.mat", mdict={'arr': Eg_signal.data})
# Plot
Eg_signal.plot(cmap="viridis", vmin = 2.6, vmax=3.8)
```

## 7. Accuracy and precision of the reconstructed band gaps

As illustrated in Supplementary Fig. S8b, the average reconstructed $E_g$ of the pure ZnO layer is approximately 3.24 eV by extracting from 260 pixels, and the standard deviation (σ) is found to be typically 0.02 eV. Compared with the directly measured map (Supplementary Fig. S8a), the reconstructed $E_g$ map retains high precision, while the average value of the band gap is somewhat higher than when measured directly. However, the increase is marginal, and we consider the reconstruction to have good accuracy in determining absolute values of the band gap. Note that for the directly measured band gap the bin-size of the histogram is related to the spectrometer dispersion, while for the reconstructed band gap a smaller bin-size is achieved due to the higher numerical precision of the Gaussian fitting to the plasmon peak.

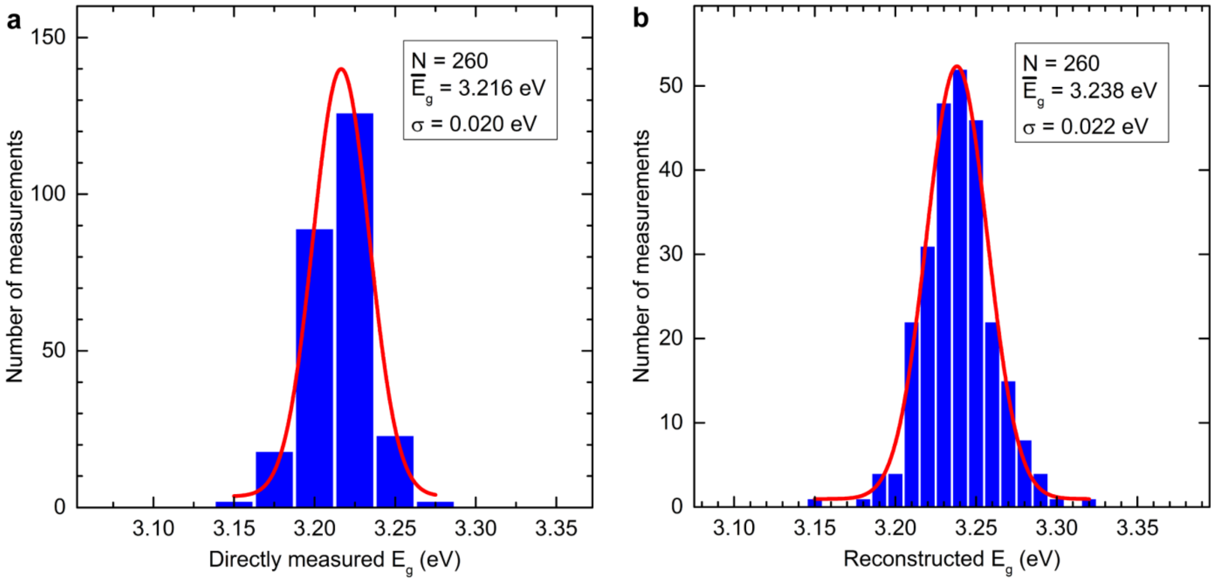


**Supplementary Figure S8.** Histogram of the (a) directly measured and (b) reconstructed band gaps of pure ZnO from the same region as Supplementary Fig. S1. A Gaussian fitting is superimposed, showing that the reconstructed band gap maps retain excellent precision, while the accuracy is slightly reduced.

## References


1. Ong, H.C., Dai, J.Y. & Du, G.T. Studies of electronic structure of ZnO grain boundary and its proximity by using spatially resolved electron energy loss spectroscopy. *Applied Physics Letters* **81**, 277-279 (2002).
2. Wu, L.L. & Zhang, X.T. Facile fabrication of ZnO:S/ZnO hetero-nanostructures and their electronic structure investigation by electron energy loss spectroscopy. *CrystEngComm* **17**, 2250-2254 (2015).
3. Zhan, W. et al. Nanoscale mapping of optical band gaps using monochromated electron energy loss spectroscopy. *Nanotechnology* **28**, 105703 (2017).
4. Granerød, C.S., Zhan, W. & Prytz, Ø. Automated approaches for band gap mapping in STEM-EELS. *Ultramicroscopy* **184**, 39-45 (2018).
5. Gu, L. et al. Mapping of valence energy losses via energy-filtered annular dark-field scanning transmission electron microscopy. *Ultramicroscopy* **109**, 1164-1170 (2009).
6. Egerton, R.F. Limits to the spatial, energy and momentum resolution of electron energy-loss spectroscopy. *Ultramicroscopy* **107**, 575-586 (2007).
7. Egerton, R.F. Electron Energy-Loss Spectroscopy in the Electron Microscope, Edn. third. (Springer, New York; 2011).
8. Schattschneider, P., Hébert, C., Franco, H. & Jouffrey, B. Anisotropic relativistic cross sections for inelastic electron scattering, and the magic angle. *Physical Review B* **72**, 045142 (2005).
9. Manoranjan, G. & Raychaudhuri, A.K. Structure and optical properties of Cd-substituted ZnO (Zn 1− x Cd x O) nanostructures synthesized by the high-pressure solution route. *Nanotechnology* **18**, 115618 (2007).
10. Wang, X.J. et al. Band gap properties of Zn1-xCdxO alloys grown by molecular-beam epitaxy. *Applied Physics Letters* **89**, 151909-151909-151903 (2006).
